# Hints and Improvements in the Practice of Medicine and Surgery

**Published:** 1799-06

**Authors:** 


### HINTS AND IMPROVEMENTS

### IN THE PRACTICE OF

### MEDICINE AND SURGERY.

ON the ſubject of *Digitalis in the Cure of Conſumption*, as mentioned in our laſt, p. 289, & ſeq. we extract the following intereſting paſſages, from a letter of a Correſpondent, dated Enfield, May 6th, 1799.

" I have had opportunities of obſerving effects, both ſalutary and injurious, very much reſembling thoſe of digitalis, in a ſtrong decoction of *green broom*, when drank in large quantities, which is, I believe, well-known to be an efficacious remedy for *dropſy*, particularly *anaſarca:* and hence it may not be unreaſonable to infer, that this plant may afford one inſtance of an analogous, ſafer, and more manageable remedy, in the treatment of conſumption.

" I was further informed, about five years ſince, that an empirical practitioner, who had acquired wealth and reputation by curing conſumptions which had been given over by the faculty, made uſe of the juice of the lake-weed, or *ranunculus aquaticus*, both as an emetic, and (in nauſeating doſes) as an alterative. His patients were ordered to drink a pint or more of water-gruel, previous to the exhibition of the juice, and then to ſwallow one ounce, or even two ounces. It generally operates expeditiouſly; but at all events, the patient was deſired to continue drinking water-gruel, at intervals, till it did operate. The gruel previouſly drank was intended to prevent the acrid property of the ranunculus on the coats of the ſtomach.

" There is a very remarkable circumſtance attending the expreſſed juice of this plant, that it may be preſerved many years, without becoming mouldy, or undergoing any material change. I have three quarts of it now by me, which have ſtood on a ſhelf, in an open laboratory, four or five years, in common quart bottles—it has the ſmell of ſweet-wort, and is quite pure."

Our correſpondent concludes with expreſſing a wiſh, that theſe circumſtances may induce practitioners to pay ſome attention to the ranunculus aquaticus, as an herb analogous in ſome of its properties to the digitalis. As, however, we have already declared our ſentiments on this ſubject, we ſhall at preſent only refer the reader to the 292d page of the preceding Number.

As connected with the preceding subject, we shall here also extract some characteristic passages from a publication which has recently appeared, entitled, "*An Essay on Consumption,*" by Dr. Beddoes. After having informed us that his own experience has fully verified the observations of Drs. Drake and Fowler (the substance of which we have given in our preceding Number, pp. 290—294), he proceeds in the following words:

"I daily see many patients in pulmonary consumption advancing towards recovery with so firm a pace, that, I hope, consumption will henceforward as *regularly* be cured by the *fox-glove,* as ague by the Peruvian bark. Could we obtain a single *auxiliary* for fox-glove, such as we have in many instances for the bark, I should expect that not one case in five would terminate as ninety-nine in an hundred have hitherto terminated. But I believe a majority of cases will yield to simple fox-glove. It is evident that no new cases need be suffered to advance beyond the first stage, without the application of this medicine, and few into it."—p. 270 and foll.

Disclaiming any share *in this most beneficial discovery,* Dr. B. says:

"*The least considerate must perceive, that if the subsequent harvest correspond to the first-fruits, there is a cause for* National Rejoicing, *greater and more universal than has ever before occurred.*"—p. 271 and foll.

---

Dr. Selig, a German practitioner, has lately published in '*Hufeland's Journal of the Practice of Medicine,*' No. I. of Vol. VII. some recent and successful experiments made with a species of *fennel (wasser-fenchel)* in the cure of *Consumption,* accompanied with further remarks on the same subject, by the editor:—in a future Number we shall communicate to our readers the result of these trials.

---

### *On the most effectual Remedies in Rheumatic Affections.*

THERE are rheumatic epidemics in which the diseases, although they derive their origin from the same source, exhibit so different and diversified a form, that it requires the sagacity of a very attentive observer, to discover their common origin, their corresponding nature, and consequently to ascertain the most accurate indications of cure.

Upon the whole, it deserves to be remarked, that the effect of the translated rheumatic matter *(metastasis)* may be extremely different, according as it is of an acrid, inflammatory, or phlegmatic nature; according to the constitution of the whole body, and the individual condition of the part affected. This matter, or humour (for what else can it be called by the nervous and chemical pathologist?) generally settles on that internal part of of the body, which has previously been weakened, either naturally, or by disease, or by other accidental circumstances. Hence we find that rheumatism principally attacks such external parts as have been in a certain degree debilitated by contusions, wounds, ruptures, dislocations, sprains, &c. Hence also it happens, that such individuals are sensible of every change of the atmosphere which affects those parts, and which as it is ludicrously said, converts their bodies into living barometers. To the same or similar causes it must be ascribed, that in certain anomalous fevers the diseased matter sometimes settles on those parts which formerly were subject to rheumatisms or erysipelatous affections, and that the period of the disease is in this manner determined: such, therefore, may be aptly called critical rheumatisms.

The most effectual method pursued in the cure of chronic rheumatism, whether arising from a venereal taint in the constitution, or other causes, is that recommended by Professor Cerillo, of Naples. It principally consists of the following simple mercurial ointment, half a drachm of which is to be rubbed in, on the sole or soles of the feet, every evening previous to going to bed:

R. *Merc. subl. subtiliss. pulv. drachm* i.

*Axung. porc. Unc.* i.

*Teratur. per hor. un. et dimid ut f. Unguent.*

The efficacy of this remedy we find recorded in the '*Journal de Medicine.*' tom. LIX; in Dr. *Richter*'s *Chirurgical Library* (in German), published at Göttingen: Vol. VII. p. 507, and 508.

According to the accounts given by Drs. Cheyne and Home, a mixture consisting of two drachms of spirit of *turpentine* and one ounce of honey, two tea-spoonfuls of which were taken every morning and evening, had an uncommon effect in promoting the discharge of urine, and relieving, in a few days, a patient who had been afflicted for near a twelvemonth, with that species of rheumatism termed *ischias.*—But Dr. Vogel, of Rostock, an eminent writer and practitioner, observes, that turpentine will relieve only that particular kind of pain before alluded to, and be of no avail in any other species of rheumatic affection. Nor does it always operate as a diuretic, and yet afford relief: sometimes however it is attended with no beneficial effects. He further remarks, that the extract of the *aconitum*, with the proper addition of *camphor*, in progressive doses, have *uniformly* proved successful in Germany; and that Dr. Herz, a respectable physician of Berlin, in one case, increased the dose of the *aconitum, even to half a drachm!* a case which almost terminated fatally; hence the necessity of attending to a certain *maximum* for a dose, which ought never to be exceeded, without the greatest precaution.

In the *nervous* ischias, another foreign practitioner, Mr. Trampel, strongly recommends the use of pills made of *sulph. antim. aur.* and *extr. opii*, in due proportions, to be increased to such a dose as the patient can conveniently bear, and until all the pains have subsided.

Another very remarkable and instructive observation relative to the treatment of rheumatism we cannot withhold from our readers, as it is registered in '*Vogel's Practical Manual*', second edit. (in German) Vol. iii. p. 447; and in '*Baldinger's New Magazine*', Vol. x. No. 2. p. 170.—Singular as it may appear to the superficial observer, it cannot be denied that the following process is founded on the established laws of the animal economy. It merely consists in gently beating the painful part of the hip or loins with a thin piece of whalebone, regularly several times a day, and immmediately after it, covering the thigh afflicted, with bags containing warm sand. This remedy is originally derived from an ingenious interpretation of a passage in Suetonius, according to whom, the Emperor Augustus was relieved (*remedio arenarum atque arundinum*) in a similar manner.

The most important and satisfactory authors who have treated on this disease, are the following:—*Ballonius*; *Riviere*; *Morgagni*, L. iv. Ep. 57; *Huxham*; *Sydenham*, Sect. vi. Cap. v.; *Stoerck*, Ann ii; *De Haen*, Tom. iv. Cap. 4; *Van Swieten*, Tom v; *Sarcone*; *Pringle*; *Monro*; *Brocklesby*; *Home*; *Baldinger*; *Macbride*; *R. E. Vogel*; *S. G. Vogel*; *Cullen*; *Clark*; *Tissot*; *Corrunni*; *Sims*; and particularly *Stoll*, in his "*Ratio medendi*", Part iii, in the chapter intitled, '*De Natura et Indole Dysenteriæ*.'

### *On Puerperal Fever, and its Treatment.*

We have already, in a preceding article of this Number, p. 381, communicated to our readers the new theory on this subject, proposed by Dr. Brickell, of Savannah: in the present paper we shall give a concise account of the various modern opinions as well as methods of treatment lately adopted by practitioners in various parts of Europe.

Professor Walter, of Berlin, maintains that in a great number of dissections of the bodies of persons who had fallen victims to the puerperal fever, he has *never* observed an inflammation of the uterus, unless it had taken place in consequence of an unsuccessful operation of the accoucheur. He also believes, with the celebrated Hunter, that the peritonæum is in a state of inflammation, and hence accounts for the suffusion of purulent matter over the intestines.

Dr

De la Roche, a French author, considers an inflammation of the intestines as the proximate cause of the child-bed fever; but this theory is certainly unfounded, and has been completely refuted by various writers, particularly the Reviewer of De-la-Roche's book in the Universal German Library, Vol. LXVIII. p. 123, &c.

Professor Soemmering, as well as Professor Stein, of Cassel, have frequently observed in dissection, that the intestines of the bodies of women who had died of the puerperal fever, were covered with a purulent or caseous matter, similar to that noticed by Walter and Hunter; but the former of these anatomists, perhaps with some degree of injustice, treats as a ludicrous idea, what others have called in this fever, a metastasis of the milk.

The aggregate of these differences of opinion has lately been definitively settled in Professor *Selle's* "*New Contributions to Physical and Medical Knowledge*" (in German), Part III. p. 92., in which this learned author has proved by incontrovertible facts: "That the disease originates in an accumulation of corrupted humours in the abdomen, which humours have either been already separated in the form of milk, or intended by nature to be so. The causes of this accumulation may be various, but are principally an epidemic miasma, passions, sudden cold, and inflammation. In corroboration of Professor Selle's theory, Dr. Hermbstädt has proved by chemical experiments, that the fluid matter found in the cavities of the abdomen was *virtually milk.* It deserves however to be remarked, that the fat of the omentum and the mesentery, being dissolved by the febrile heat, may combine with the extravasated lymph, so as to produce a fluid of a more or less viscid consistence, and resembling milk in its external characters.

Almost all the French writers *Le Roy*, *Puzos*, *Levret*, *Delcurye*, *Paulet*, *Doulcet*, *Doublet*, as well as *Boerhaave*, and partly also *Van Swieten*, *Gruner*, *Fuchs*, and many others, consider the metastasis of the milk as the leading cause of the childbed fever. But it will appear from the following considerations, which are confirmed by daily experience, that the milk is altogether unconnected with the disease: 1, That lying-in women who suckle their own children, and have an abundant portion of milk in their breasts, are not exempt from the attacks of the fever; 2, That although the milk be scanty or deficient, sometimes neither fever nor any other distressing symptom will be the consequence; 3, That the fever with all its concomitant symptoms has frequently prevailed for several days, before the milk disappeared; 4, That the breasts in many cases remain filled with milk to the last stage of the fever; and 5, That in a variety of cases, after dissection, no no traces of a metastasis of milk could be discovered. If therefore, such metastases really occur in puerperal fever, as have been frequently observed and sufficiently demonstrated by accurate practitioners, it ought to be determined in what condition they stand with the fever. Upon the whole, they are to be considered as *symptomatic* only, and not *pathognomic*.

We have been perhaps more diffuse, in stating the different opinions that have hitherto prevailed respecting the material cause of puerperal fever, than is strictly consistent with this department of our Journal; but as false theories frequently lead to false practice, we thought ourselves justified in extending the limits of this article.

With respect to the method of cure in this fever, we shall premise one very important rule, not to attack the disease with violent remedies, but to employ those of the mildest kind, so that the attempts of the physician may not do more mischief than the disease. This remark particularly relates to evacuants, although the principal indications of cure ought not, on that account to be neglected: hence all acrid and stimulating remedies should either be avoided, or at least covered in emulsions, gum arabic, and the like, that they may fulfil the intention, without exciting too violent a commotion in the irritable body of the patient.

Phlebotomy should not be resorted to, without the most pressing necessity. Let every thing be carefully avoided that can in any degree disturb the tranquility either of mind or body. With respect to the debility accompanying this fever, a due distinction ought always to be made between hysteric weakness, and that arising from want of vital energy; and the practitioner should not be too timid in removing, as speedily as the strength of the patient and other circumstances will admit, any material cause which can be discovered, and may prove a powerful stimulus to the nervous system: for a few drops of acrid bile, or rancid mucus, may be productive of the most dreadful consequences. Hence the necessity of employing evacuants, with a steady and skilful hand; and to support the patient occasionally with other appropriate medicines, such as the castoreum, assafœtida, valerian, &c. as may best agree with her general state.

The sudorific remedies, as well as camphor, blisters, the warm bath, opium, bark, &c. which, in this fever, are frequently prescribed in a very empirical and indiscriminate manner, are each of them useful in proper time and situation, but none of them at all times, and in all cases.

A remarkable instance of the prevention of this disease occured in the hospital at Lyons, where, according to Pouteau's method, two or three emetics emetics are regularly administered to women during their pregnancy, instead of bleeding them; and this precautionary step is attended with such happy effects, that since its first introduction, the cases of puerperal fever, or other diseases peculiar to lying-in women, have become extremely rare. Indeed such a fever, since that period, is there scarcely known by name, and its having proved mortal is a thing quite unheard of. Vide '*Richter's Chirurgical Library*' (in German), vol. viii. p. 75.

The best modern writers on this subject are, *Strother*, *Hulme*, *Leake*. *White*, *Kirkland*, *Butter*, *Denman*, *Hunter*, *Manning*, *Millar*, *Aikin*. *Johnstone*. *Stoll*, *Home*, *Gruner*, *Fuchs*, *Selle*, *Burserius*, *Doublet*, *Delaroche*, *Smellie*. *Osiander*, *Reil*, *Walsh*, *Clarke*, *Ratzki*, *Kaublen*, *Thilenius*, *Nolte*, *Zehner*, &c. &c.

---

### *On the Medicinal Properties of Iron.*

Cit. Mandel, professor of pharmacy and therapeutics at Nancy, member of the central jury of public instruction, has, in a late dissertation upon iron, endeavoured to explain the different neutral states in which it is found, as well as those to which it can be converted by art; the advantages derived from its magnetic property, particularly by the discovery of the compass; and its quality of attracting and conducting the electric fluid disengaged in lightning: he further attempts to demonstrate the affinity subsisting between that metal and oxygen, from the facility with which it forms combinations with saline substances; from its ready oxydation by air and water, and its solution in this last substance which is decomposed by it

He then passes to the action of iron on the animal economy; the cures which it sometimes speedily performs, and on the other hand, the accidents it is apt to occasion in certain individuals, even when given in small doses. This circumstance tends to determine a very interesting question, namely, whether the iron which exists in substance and in form in our humours, particularly in the blood, may not be considered, on account of its natural increase or diminution, as the cause of many diseases? He mentions *chlorosis*, as one which leads to a satisfactory result.

of oxygen, rather than increafe it; that it is cured by iron which has undergone no preparation, but the minuteft divifion of its particles; whence he concludes that we ought not to confider *oxygenation*, but rather *ferrugination*, as the efficient caufe of cure.

The author next fettles the nomenclature of different diforders in which iron, or various preparations of it ought to be adminiftered; he points out thofe in which it is refpectively indicated, as far as relates to the nature and ftate of each.

Laftly, he examines, whether the *magnet* fhould be admitted in the lift of medical remedies, and enumerates the different fyftems, both of the advocates of this mineral and its opponents: he combats, from experience, the opinion of the latter, who neverthelefs admit deleterious fubftances; he fhews that it is at leaft doubtful, whether the iron contained in the blood be not attracted by the magnet; and, without admitting the truth of all the wonders attributed to it, and at the fame time making allowances for the extravagance of the praifes lavifhed on it, he is of opinion that it ought to be preferved among the number of medicinal remedies.

We have purpofely extracted this imperfect account from the xxviith. Number of the 'Recueil Periodique de la Société de Médecine de Paris;' to give our readers a fpecimen of the manner in which articles of a practical tendency are generally treated in foreign Journals. Not a word of the dofes, or particular cafes, in which either the *iron* or the *magnet* can be adminiftered with fafety! In a future Number of this Journal, we propofe to refume this fubject, and to fupply thefe deficiences.

### *On Bleeding Children afflicted with a large Head.*

In the firft volume of the Memoirs publifhed by the National Inftitute of France, we met with an interefting Effay of Cit. Desessartz, "*On the advantage and neceffity of taking but little blood at a time, from children afflicted with a large head.*" This memoir is avowedly the refult of numerous obfervations, from which the author has felected two, as the moft decifive. Thefe, as well as the general tenor of the work, tend to fhew:

1. That children with large heads, that is to fay, thofe in whom that part of the body is of a greater fize than is confiftent with a found ftate of health, or the juft proportions of nature, have the leaft energy, and are moft fubject to convulfions.

2. That taking half a cup-full (palette) of blood, from the age of fix months to a year, a cup-full and a half, or two cups-full, from the age of one to to ſix years, are too copious; and that topical bleeding by means of leeches may be ſubſtituted with advantage, producing a more gradual, and leſs debilitating evacuation; and

3. That ſuitable drinks, bathings, and above all emollient fomentations of the belly may be employed with great ſuccefs as auxiliaries.

Cit. Deſeſſartz conſiders theſe means united to be the foundation of the proper treatment of many other diſeaſes of ſuch children as have preternaturally large heads. Reſpecting theſe, and indeed children in general, the aphoriſm of Hippocrates, on the facility of accompliſhing a criſis, is verified, when the phyſician has been fortunate enough to overcome the firſt obſtacles, and thus to prevent the regular developement of the phenomena exhibited by the diſeaſe.

---

### *On the propriety of performing the Cæsarean Operation.*

THE Medical Society of Paris, after having heard the Memoir of Citizen Baudelocque, on the Cæſarean Section, read twice, together with the intereſting diſcuſſion it has produced; and conſidering

1. That it is proved by experience, that there are caſes in which delivery is impoſſible by natural means;

2. That, in a number of caſes, the Cæſarean Operation is the only means which affords any hope of ſaving the life of the child; and

3. That this operation, ſerious as it is, has been *often* practiſed with complete ſucceſs—

" Are unanimouſly of opinion, that it is the duty of the practitioner to have recourſe to the Cæſarean Operation in thoſe caſes where his profeſſional ſkill deems it proper: and in order to enable men of ſcience, as well the public, to judge of an operation ſo intereſting to humanity, *ſocial order*, and the progreſs of the art, the ſociety decrees, that Citizen Baudelocque's memoir containing his inquiries and reflections relative to this ſubject, ſhall be printed and diſtributed among the different Adminiſtrative and Judicial Bodies."

" Without adding any commentary to this ſingular reſolution of the Medical Society of Paris, we ſhall only refer the reader to ſome former articles on the ſame ſubject, which he will find in No. II. p. 197, and No. III. p. 308 and 309 of our Journal. It would be needleſs, and inconſiſtent with our plan, to ſay more reſpecting this controverſy which is now at iſſue before the public, and which has been diſcuſſed with equal calmneſs and ſcientific judgment in this country.

